# “In my day…”- Parents’ Views on Children’s Physical Activity and Screen Viewing in Relation to Their Own Childhood

**DOI:** 10.3390/ijerph15112547

**Published:** 2018-11-13

**Authors:** Emma Solomon-Moore, Lydia G. Emm-Collison, Simon J. Sebire, Zoi Toumpakari, Janice L. Thompson, Deborah A. Lawlor, Russell Jago

**Affiliations:** 1 Centre for Exercise, Nutrition & Health Sciences, School for Policy Studies, University of Bristol, 8 Priory Road, Bristol BS8 1TZ, UK; E.Solomon-Moore@bath.ac.uk (E.S.-M.); Simon.Sebire@bristol.ac.uk (S.J.S.); Z.Toumpakari@bristol.ac.uk (Z.T.); Russ.Jago@bristol.ac.uk (R.J.); 2 School of Sport, Exercise and Rehabilitation Sciences, University of Birmingham, Birmingham B15 2TT, UK; J.Thompson.1@bham.ac.uk; 3 MRC Integrative Epidemiology Unit at the University of Bristol, Oakfield House, Oakfield Grove, Bristol BS8 2BN, UK; D.A.Lawlor@bristol.ac.uk; 4 Population Health Science, Bristol Medical School, University of Bristol, Canynge Hall, Whiteladies Road, Bristol BS8 2PS, UK

**Keywords:** childhood, physical activity, screen viewing, independent mobility, generations, qualitative

## Abstract

Physical activity and screen viewing are associated with cardio-metabolic risk factors, psychological wellbeing, and academic performance among children. Across the last generation, children’s physical activity and screen viewing behaviours have changed, coinciding with changes to the home and neighbourhood environment. This study aimed to qualitatively explore parents’ views on their 8–9-year-old child’s childhood and how this compares to experiences from their own childhood, with a specific focus on physical activity and screen viewing behaviours. Semi-structured telephone interviews were conducted with 51 parents (mean age = 41.2 years, range 31.5 to 51.5 years), between July and October 2016. Inductive and deductive content analyses were used to explore parents’ perceptions of their child’s physical activity and screen viewing behaviours in comparison to their own childhood behaviours. Interview data revealed that compared to the relative freedom they recalled as children, parents restrict their children’s independent mobility and outdoor play due to concerns about safety. Despite their children having greater access to structured activities than they did as children, parents feel their children are “missing out,” and perceived their own childhood as better with regards to maximising independent and outdoor play and limiting screen viewing. Innovative strategies are needed to change the social norms surrounding children’s independent mobility and outdoor play.

## 1. Introduction

Physical activity and screen viewing (e.g., watching television, using computers, mobile phones, tablets, and playing video games) are modifiable behaviours associated with a number of cardio-metabolic risk factors [1,2], psychological wellbeing, and academic performance among children [3,4,5]. Data from the nationally-representative Millennium cohort showed that only 51% of 7–8-year-old children met the Chief Medical Officer’s recommendation of an hour per day of moderate-to-vigorous-intensity physical activity [6,7], while data from the East of England Healthy Hearts study showed that 36% of 10–15-year-olds engaged in over two hours of screen viewing per day [8]. Despite efforts to increase physical activity and manage screen viewing among children [9,10,11], the limited data available are inconclusive when exploring trends in these behaviours over the last few decades, with the majority of studies focusing on specific contexts (e.g., organised sport, active transport, television viewing), reporting mixed results and inconsistent magnitudes of change [12,13], with some evidence suggesting that children today are less active than they were a generation ago [14,15]. Therefore, more research is needed to understand how these behaviours have changed across time to find ways to increase physical activity and manage screen viewing among children.

Evidence suggests that children’s independent mobility and outdoor play have decreased in recent years [13,16]. Long-term trend data from England indicates that, in 1971, 86% of primary school children (aged 4–11 years) travelled home from school without their parents, whereas in 2010 this figure was 25% [17]. One potential explanation is that children are living further distances from schools, with one UK study finding the mean school travel distance for children aged 11–16 years increased from just over two miles in the mid-1980s to almost 3.7 miles in 2013 [18]. Independent mobility and outdoor play are associated with overall physical activity among children [19,20,21,22], as well as with increases in children’s environmental and social knowledge and competence, feelings of self-worth, efficacy and resilience, and reductions in anxiety [23,24,25,26,27,28,29]. However, both children and parents express concerns about children being outdoors [30], with parents generally reporting being more concerned than children [31]. Safety, traffic, and stranger danger have all been cited by parents as reasons for lower levels of independent mobility and outdoor play among children [32,33,34,35,36,37], although these fears of danger generally outweigh the realities of neighbourhood crime and safety [36,38].

There is consistent evidence that contemporary parents value children’s time outside [16]. However, parents’ fear of danger has resulted in a generation of ‘bubble-wrapped’ children whose time spent outside is often structured and supervised by adults [27,39,40]. This has led to a cyclical relationship whereby parents drive children to most destinations instead of allowing them to walk or cycle [39,41], resulting in more cars on the road and less people walking (‘natural surveillance’; [42]), thus roads appear more dangerous and safety concerns are exacerbated [27,43]. Furedi has termed this a ‘culture of fear’ [44], whereby safety fears are perpetuated by the media [40,45] and parents create new folk stories of terrorism, drug gangs, drive-by shootings, and dangerous men in order to keep children inside [27]. Despite the declines in independent mobility and outdoor play, a wider range of outdoor and indoor children’s activities have emerged, and many children have more opportunities to engage in structured after-school activities than before [16].

Many parents recognise their children’s outdoor play as different from their own experience [36]. A generation ago, playing meant playing outside ‘all the time’, regardless of weather, and the public street space belonged to children [43]. Compared to previous generations, most children’s bedrooms now offer numerous play possibilities, in part due to the increasing size and number of bedrooms in households, as well as the availability of toys and screen viewing devices [46]. Children’s bedrooms are considered to be both a safe space by parents and an escape from parental control by children [47]. Despite the numerous play opportunities afforded to many children in their own homes, for some children ‘play’ indoors revolves around watching television or engaging in other forms of screen viewing [48]. Since its inception, television viewing has always been extremely popular; however, a generation ago households tended to have only a single television, the number of channels was limited, children’s programmes were broadcast much less frequently, and on-demand streaming services were not available. Technology now features highly in modern homes, with a larger variety of media devices available, and more children owning personal devices (e.g., televisions, computers, games consoles, tablets, and mobile phones) than ever before [49]. Therefore, we need to understand how these changes in screen viewing device availability and the ways children engage in screen viewing have an influence on their childhood experiences, and how this compares to children’s screen viewing experiences a generation ago.

The purpose of this qualitative study was to explore mothers’ and fathers’ perspectives on their 8–9-year-old’s childhood in relation to how this compares to experiences from their own childhood, with a specific focus on their physical activity and screen viewing behaviours. To date, no studies have examined this topic. There is a paucity of information on physical activity and screen viewing behaviours of children during the middle primary (elementary) school years, with most studies focusing on the start or end of primary school. This gap is particularly important, as children’s physical activity levels progressively decline during primary school [50,51,52] and this age group is on the cusp of greater independence but still reliant on parental supervision and control [53]. Due to the changes in children’s physical activity and screen viewing behaviours across the last generation that have coincided with changes to the home and neighbourhood environment [13,46,49], it is important to understand parent’s views on the physical activity and screen viewing environment their children are growing up in, and how that compares to their views of their own childhood. There is a tendency in society to assume things were better in the past, and so it is important for us to contextualise how childhoods have evolved across the last generation, to understand in what ways things were better, what elements are new, what belongs in the past, and how these changes have influenced behaviour. In this way, we can learn more about where parents feel there is scope or desire for behaviour change, which will enable us to develop strategies to help parents promote a healthy balance of physical activity and screen viewing time for their children.

## 2. Materials and Methods

The current study used data from the longitudinal B-Proact1v study, which has been described in detail elsewhere [50,54,55]. Briefly, the study aimed to examine factors associated with children’s and parents’ physical activity, sedentary time, and screen viewing behaviours. In 2012–2013, data were collected from 1299 5–6-year-old children and at least one of their parents, from 57 primary schools across Bristol, UK. Between March 2015 and July 2016, 47 of the original schools were re-recruited when the children were 8–9 years old, and data were collected from 1223 families. Children and parents wore a waist-worn ActiGraph wGT3X-BT accelerometer for five days, including two weekend days. ActiGraph accelerometers have been demonstrated to provide valid assessments of physical activity in laboratory and free-living conditions [56,57]. Accelerometer data were processed using Kinesoft (v3.3.75; Kinesoft, SK, Canada), and were included if at least three days of valid data (including at least one weekend day) was provided. Valid days were defined as at least 500 min of data after excluding intervals of ≥60 min of zero counts, allowing up to two minutes of interruptions. Minutes spent in moderate-to-vigorous-intensity physical activity (MVPA) and mean sedentary time (SED) per day were derived using population-specific cut points for children [58] and adults [59]. Children’s height, weight, and blood pressure were also measured.

**Figure 1:**
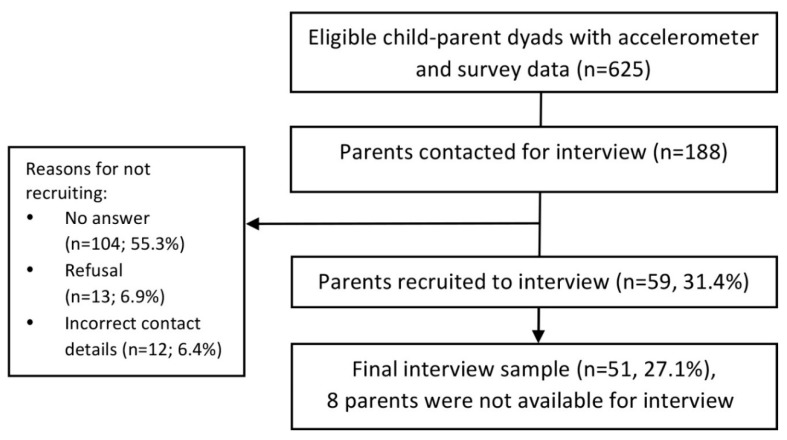
Study flow of participants.

Between July and October 2016, semi-structured telephone interviews were conducted with a sub-sample of 51 parents who participated in the study at Year 4. Telephone interviews were used as the method for data collection, because they provide a cost-effective way of collecting information and allow flexibility for both the participant and the researcher [60]. Only families with complete data for all measures (accelerometer and questionnaire data, child height, weight, and blood pressure) were eligible for inclusion in the interview sample (*n* = 625). This sample was stratified according to the child’s MVPA minutes per day (dichotomised around the median: 57.5 min), SED minutes per day (dichotomised around the median: 434.6 min), and by child gender. This produced eight sub-groups (1 = low MVPA, low SED boys; 8 = high MVPA, high SED girls). The order in which parents were invited to participate in an interview was randomised within each sub-group. One hundred and eighty-eight parents (30%) were contacted, of which 59 (31.4%) agreed to participate in an interview, and 51 (27.1%) completed an interview (Figure 1). Interviews were audio-recorded using an Olympus DS-3500 encrypted digital recorder, and continued until theoretical saturation was reached for the entire sample through the repetition of responses. Interviews were conducted at the interviewee’s convenience; 37 during weekday daytimes (72.5%), 13 during weekday evenings (25.5%), and one on a weekend evening (2%). Participants received a £10 high street shopping voucher as a thank you for their time. The study received ethical approval from the School for Policy Studies Ethics Committee at the University of Bristol, and written parent consent was received for all participants.

### 2.1. Interview Data

The study was rooted in a realist ontology, whereby there is “the belief that there exists a reality out there, driven by immutable natural laws” [61] (p. 19). A semi-structured interview guide was developed and refined by the research team, informed by gaps in current knowledge, and guided by the baseline B-Proact1v quantitative and qualitative findings [54,55,62,63,64]. The questions explored a variety of topics, including parents’ perceptions of their 8–9-year-old child’s physical activity and screen viewing behaviours, their understanding of what has changed regarding these behaviours since their children were 5–6 years old, and parents’ physical activity and screen viewing experiences during their own childhood in relation to their child’s experiences. Example questions from the interview guide included ‘Compared to other children their age, how active do you feel your child is?’, ‘Compared to experiences from your own childhood, how do you feel that physical activity is different for your child now?’, and ‘Looking back across the last three years, what has changed with your child—in terms of their screen viewing?’. Questions were posed in a non-leading manner to allow participants to shape the direction of the interview, and issues that emerged were probed with further questions. Interviews were conducted by two female researchers who were trained in conducting qualitative interviews. The interview guide was informally piloted with a small sample of parents to test the wording of questions and adjustments to the interview guide were made accordingly. Researchers met regularly throughout data collection, approximately every five interviews, to discuss the suitability and order of the interview questions and make minor alterations to the interview guide.

### 2.2. Demographics

Parents provided parent and child gender, date of birth, ethnic origin, and parental employment via a questionnaire. Where children’s date of birth was missing (21% of children), and considering all children were in the same school year with a maximum age range of 12 months being legally possible, the median age of 9.0 years was assigned. As an indicator of socio-economic status, an Index of Multiple Deprivation (IMD) score based upon the English Indices of Deprivation [65], was assigned to each child based on their reported home postcode, where a higher score indicates a greater level of deprivation.

### 2.3. Data Analysis

Interviews were transcribed verbatim and anonymised before being entered into QSR NVivo 10 (QSR International, Warrington, UK) to facilitate analysis. Interview data were analysed using the Framework Method [66], which outlines a systematic method of categorising and organising qualitative data to produce highly structured outputs of summarised data. Following the Framework Method, analysis involved several phases: familiarisation, coding, framework development, framework application, and interpretation. During familiarisation, transcripts were thoroughly read and re-read independently by two researchers to immerse themselves in the data. After familiarisation, each researcher independently read the transcripts line by line and applied ‘codes’ to describe important passages in the text. An initial coding frame was then developed collaboratively by the researchers, applied to the data, and refined throughout the analysis process. Thematic content analysis enabled themes to develop both inductively from the accounts (experiences and views) of participants, and deductively based on pre-existing ideas from existing literature [67,68]. In terms of the inductive process, codes were developed to reflect new themes that emerged from the interview discussions, such as the influence of the built environment. With respect to the deductive process, some codes were developed based on the interview questions, such as the value placed on physical activity. The two researchers met regularly to ensure accuracy and consistency. Any disagreements that occurred during coding were discussed with additional members of the research team to ensure consensus, and no disagreements remained unsolved. Hierarchies of themes were created and summarised, and brief summaries, mind maps, and representative quotes for each category were extracted for reporting purposes. Final quotes were selected to be illustrative of several responses given by participants. For quantitative data, means and proportions were used to describe participants. Parents’ MVPA and SED were dichotomised around the median values (45.5 min and 532.2 min, respectively). To increase understanding about the child and parent relevant to each quote, information on interview number, child and parent gender, classifications (low, high) of physical activity and sedentary time, and household IMD score are provided after each quote.

## 3. Results

Table 1 presents the characteristics of the interview participants and their children. Thirty-one participants were mothers and 20 were fathers, with an average age of 41.2 years (range 31.5 to 51.5 years), and 94.1% were White British. The mean score for household deprivation was 11.5 (range 1.6 to 44.5), which sits within the second-least deprived quintile group [65]. The average interview duration was 34.4 min (SD: 8 min, range: 18 to 55 min). Parents compared their child’s physical activity and screen viewing to their own childhood behaviours, and were divided regarding how their child’s physical activity levels compared to their own as children. Some parents felt that their child is more physically active than they were, because children now engage in more organised sports and schools provide more opportunities for physical activity than during their childhood. Some parents felt that activity levels were similar, with only the types of activities differing between childhoods, while other parents felt they were more physically active when they were children because they engaged in more free play and spent more time outdoors. In contrast to the divergent perspectives on physical activity, quite a few parents expressed that their child engages in more screen viewing than they did, primarily due to a lack of choice for screen viewing when they were young. Only one parent suggested that their child engages in less screen viewing, stating that they did not have screen viewing rules when they were young, but they have rules for their children now.

**Figure 2:**
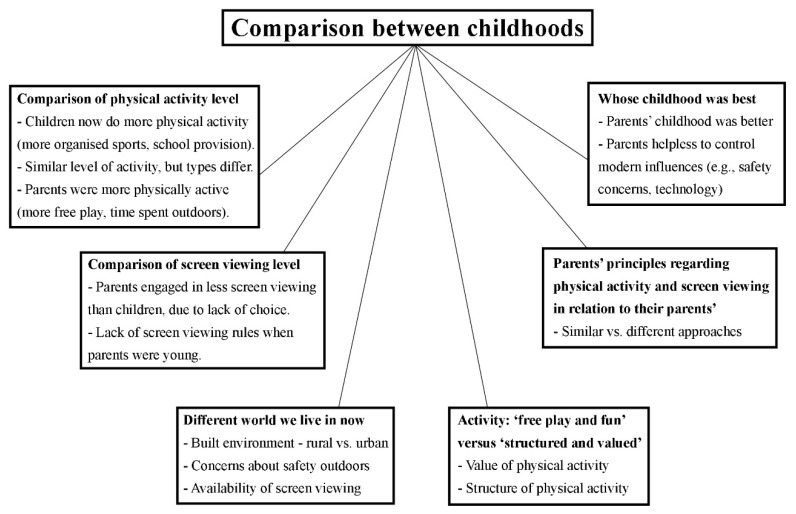
Presentation of the main themes and subthemes.

Four main themes emerged from the data: (1) the different world we live in now; (2) activity—‘free play and fun’ versus ‘structured and valued’; (3) parents’ principles regarding physical activity and screen viewing in relation to their parents’; and (4) whose childhood was best (Figure 2).

### 3.1. The Different World We Live in Now

Many of the parents discussed how the world we live in now in is different from the world they experienced growing up, in terms of the built environment, concerns about safety outside, and the availability of screen viewing.

#### 3.1.1. The Built Environment

Numerous parents described how the environment their child is growing up in is different from where they grew up. Some parents described living in rural areas when they were children and so they had more space to play, which resulted in them doing more physical activity. On the contrary, other parents perceived that growing up in the city encouraged them to play out more because their friends were closer and it was easier to walk places. It appears that as time has gone by, both environments (rural and urban) are different now from what they once were, which has an influence on physical activity behaviours of children.

“There isn’t anywhere near the amount of outdoor space because he’s in a city and I was in a village in the countryside, so I think there was a lot more space and loads more room to run, play games.”

(Int 43; Child: boy, low MVPA, high SED; Parent: male, high MVPA, low SED; IMD score: 10.2)

“I suppose we did walk a bit more ‘cause I used to live in the middle of a city so you could walk… Whereas where we live, there isn’t even a path along the main road so you couldn’t safely walk, we can walk over the fields but, we couldn’t walk to a local park or we’ll have to drive... We can’t walk to school, it wouldn’t be possible.”

(Int 36; Child: boy, low MVPA, low SED; Parent: female, low MVPA, high SED; IMD score: 10.5)

Among these parents, fathers and mothers were equally represented, deprivation levels were very similar to the overall group, and parent activity tended to be classified as ‘low MVPA, high SED’, while child physical activity and sedentary time was representative of the wider group.

#### 3.1.2. Concerns about Safety Outdoors

Several parents described safety concerns regarding their child playing outside or being able to walk to the local park, citing the greater number of cars on the road or ‘stranger danger’ as reasons. Parents reported feeling a responsibility to ensure that they, or another adult, supervise their children when they go out or else they may be considered a bad parent. It is now normal for parents to be fearful and cautious, and it is not the norm to let your children ‘knock for a friend’. Some parents expressed guilt about their fears, as they would prefer to allow their child more freedom.

“We never got driven to school, we walked to school, and we walked to school on our own a lot of the time, we can’t do that now really because at the moment, society the way that it is or at least perceived, you don’t send your child out in the morning to go and knock on a friend’s door and spend the day running around the town, and if you do you’ll be considered a bad parent even if you think it’s okay.”

(Int 49; Child: girl, high MVPA, high SED; Parent: male, low MVPA, high SED; IMD score: 8.1)

“We were allowed to wander off and go off on our bikes and I think there wasn’t as much fear as there is now. It’s certainly a huge thing as a parent. We’re all guilty of that now. I think we have this vision and if we let them go anywhere they’re gonna be kidnapped and goodness knows what, because it goes on and I suppose logically we know it’s highly unlikely. But on the whole, roads are a lot busier. Everything’s a lot busier. Certainly, at 9 years old I wouldn’t let [child] go off with a friend on her own, so if she wants to go off on her bike or her scooter I go with her or take them to the park and supervise.”

(Int 9; Child: girl, high MVPA, high SED; Parent: female, low MVPA, high SED; IMD score: 13.8)

“I suppose it is the safety aspect as well, because you know where your kids are, they’re in their bedroom, they’re safe and they’re not out on the streets.”

(Int 18; Child: boy, high MVPA, low SED; Parent: male, low MVPA, high SED; IMD score: 13.9)

A greater proportion of mothers than fathers reported safety concerns; however, similar reasons were provided by both genders. Parents who expressed concerns about their child’s safety outdoors were slightly more deprived than the overall group, their activity tended to be classified as ‘low MVPA, high SED’, and they were representative of the wider group in regard to their child’s activity behaviours.

#### 3.1.3. Availability of Screen Viewing

Nearly all parents mentioned the large availability of screen viewing devices now versus the lack of choice for screen viewing when they were children, whereby screen viewing primarily consisted of television, with limited channels, and so the default option was to go out and play. Several parents equated more media devices to less physical activity, while others suggested that technology has made life easier so children are lazier and don’t have to think as much. In contrast, a few parents felt that screen viewing doesn’t have an impact on children’s behaviour because children have grown up around technology and thus do not know any different.

“Whereas now it’s on every device possible. We’ve got a TV in every room, so it’s just more normal for the children to do that rather than play I think…he definitely does [screen viewing] more.”

(Int 22; Child: boy, low MVPA, high SED; Parent: female, high MVPA, low SED; IMD score: 12.3)

“So I think there’s probably greater opportunities for them, there’s also greater distraction, children’s television was something that was a couple of hours a day when I was a child, not 24/7.”

(Int 42; Child: boy, high MVPA, high SED; Parent: male, low MVPA, high SED; IMD score: 1.7)

“I think now, everything’s so readily available that it’s almost like watching the telly on the computer some of the time—and in an awful lot of games or even things to help with homework, they’ve not really got to do an awful lot of thinking. I think with us, the technology was so poor in comparison to how it is now, that everything that you did, you had to think about.”

(Int 31; Child: girl, high MVPA, low SED; Parent: female, low MVPA, high SED; IMD score: 2.6)

“Literally, you need to have a laptop now as much as you had to have a slate in 1800s so it’s just the way it is.”

(Int 49; Child: girl, high MVPA, high SED; Parent: male, low MVPA, high SED; IMD score: 8.1)

“Well, I don’t know if its [screen viewing] had any impact because they don’t know any different, do they? It’s not like they’ve come from that time and they’ve got knowledge of that.”

(Int 18; Child: boy, high MVPA, low SED; Parent: male, low MVPA, high SED; IMD score: 13.9)

The large availability of screen viewing devices now compared to the relative lack of devices when they were children was mentioned by similar proportions of mothers and fathers. This group was slightly more deprived than the overall group, was representative of the overall group for parent activity, and tended to have children with activity levels classified as ‘high MVPA’ and a mix for sedentary time.

### 3.2. Activity: ‘Free Play and Fun’ vs. ‘Structured and Valued’

Parents perceived that children’s physical activity is conceptually different now to how it was when they were children, in terms of the value placed on physical activity and how structured activities are.

#### 3.2.1. Value of Physical Activity

The majority of parents perceived that when they were young physical activity was just about having fun, and nobody talked to them about the values of being active. Alternatively, most parents believed that, nowadays, children are conscious of the valuable aspects of physical activity (e.g., “keeps you fit”, “good for your heart”, “stops you getting fat”, and “you live longer”), which are often emphasised by parents and schools, as well as general messaging through the media. Only a few parents mentioned that they were aware of the value of physical activity when they were children.

“I think it [physical activity] was just having fun. I don’t think anybody used to talk to us about, ‘you need to be fit’ and, ‘you need to be doing exercise,’ and stuff. I don’t think that was even talked about when I was young.”

(Int 22; Child: boy, low MVPA, high SED; Parent: female, high MVPA, low SED; IMD score: 12.3)

“I think they’re more aware that it’s [physical activity] important. I think there’s more messaging around these days about, it’s really important that you do exercise, it’s really important that you stop watching your computer and go outside and play on the trampoline for a bit. I think there’s a lot of messaging around about that.”

(Int 5; Child: boy, high MVPA, high SED; Parent: female, low MVPA, high SED; IMD score: 7.8)

Parents who discussed how awareness of the value of physical activity has increased since they were young were representative of the wider group for parent gender, deprivation, and parent physical activity, but tended to have children with activity levels classified as ‘high MVPA, high SED’. The parents who suggested they were aware of the value of physical activity when they were children tended to be mothers, less deprived than the group average, moderately active, and have children whose activity was classified as ‘low MVPA, high SED’.

#### 3.2.2. Structure of Physical Activity

Most parents felt that children’s physical activities are more structured now, in the form of after-school clubs, and that there is more provision for children’s activities now compared to when they were growing up. Parents also believed that they engaged in more free play and spent greater time outdoors as children, considering physical activity to be an integral part of their daily routine, through walking, playing outside, and/or doing manual chores. Parents generally equated time spent outside with physical activity. However, several parents felt that their child engages in similar activities compared to their own childhood.

“I think we were all encouraged, 30 years ago, to just go out and play and be children whereas now it’s far more regimented and, kids go to school, we then all ferry our children to their million clubs and then home, tea, shower, bed.”

(Int 14; Child: girl, high MVPA, low SED; Parent: female, high MVPA, low SED; IMD score: 7.0)

“There wasn’t anything like that around then [places for activities], that was unheard of, so you were doing things that were either already there like going fishing on the canal, playing football on the grass near your house, and you just made of it what you could with what was there. It wasn’t a purpose-built centre for activities, it was just a local green space with two trees for goals and you just got on with it.”

(Int 16; Child: girl, low MVPA, low SED; Parent: male, high MVPA, low SED; IMD score: 2.5)

“A lot of her [child] sport is associated with the school, and it seems that they are available, and also obviously they are free. So, that’s really positive, whereas when I was at school we didn’t have any of that. You finished at 3:15 and you went home. There was none of this extracurricular activity stuff.”

(Int 24; Child: girl, low MVPA, low SED; Parent: female, low MVPA, low SED; IMD score: 4.1)

“I think as far as the activities are concerned, what’s on offer with school and she’s just done her cycling proficiency and things like that, I think that’s all still the same.”

(Int 13; Child: girl, low MVPA, high SED; Parent: female, high MVPA, low SED; IMD score: 2.1)

Compared to the overall group, parents who felt that children’s activity is more structured nowadays were representative for parent gender, tended to be less deprived, were more likely to be classified as ‘low MVPA’ for parent activity and ‘high MVPA, low SED’ for child activity. Parents who felt activities were similar between childhoods tended to be mothers, less deprived than the overall group (but similar to parents who felt children’s activity is more structured now), with activity levels tending to be classified as ‘low MVPA, high SED’ for parents and ‘low MVPA’ for children.

### 3.3. Parents’ Principles Regarding Physical Activity and Screen Viewing in Relation to Their Parents’

Several parents discussed their own parents’ principles regarding screen viewing and physical activity from when they were growing up. Some participants felt that their parents’ approach to these behaviours was appropriate, using phrases like “we turned out okay”, and so they have utilised similar approaches for their children. Alternatively, others mentioned that they use different approaches with their children compared to those they experienced during childhood, both in terms of physical activity and screen viewing.

“I mean in the evening the TV seems to be generally always on. And it does seem to be the default kind of relaxation tool for, well probably everyone in the family. And that’s the same for me and my parents when I was growing up so I think the TV is the kind of the tool in the corner that is permanently on these days, it’s a permanent fixture really.”

(Int 19; Child: girl, low MVPA, low SED; Parent: male, high MVPA, high SED; IMD score: 10.5)

“I dunno, subconsciously we probably think what our parents did was probably just about right, we turned out okay, so I let my kids watch as much TV as I did.”

(Int 38; Child: boy, high MVPA, high SED; Parent: male, low MVPA, high SED; IMD score: 8.6)

“It’s just that was my particular childhood, I stayed inside a lot, and would have liked to have gone outside a lot more and played outside so I try to encourage my kids to do that more.”

(Int 33; Child: girl, low MVPA, high SED; Parent: female, low MVPA, low SED; IMD score: 4.8)

“I watched quite a lot of telly when I was younger, the telly just used to be on, and we used to just watch what you want and so I almost don’t want it to be like that.”

(Int 35; Child: girl, high MVPA, high SED; Parent: female, low MVPA, high SED; IMD score: 2.1)

Parents who reported recreating their parents’ approach to managing physical activity and screen viewing tended to be more deprived than the wider group. In contrast, parents who reported using different approaches to managing behaviours compared to their parents were less likely to be deprived. There were no differences between the activity sub-groups for either child or parent activity.

### 3.4. Whose Childhood Was Best

Generally, parents perceived their childhood to have been better than their children’s and reported not liking the way that their child’s behaviour has been influenced by safety concerns, technology, and the increased structuring of children’s lives. Parents suggested they were helpless to control these influences, but do what they can to ensure their children have similar childhood experiences. Some parents even expressed feelings of sadness because their children cannot experience their childhood.

“It would be nice to go back to, you know, when—to let them sort of experience what we had in a way.”

(Int 18; Child: boy, high MVPA, low SED; Parent: male, low MVPA, high SED; IMD score: 13.9)

“I know it’s completely different these days, but I do try for them both to have the childhood that I did, I like them to be out, if only out in the street nine times out of 10, so, they are literally out there until the lights come on and then they’re in. So, I do try and follow my childhood because it was it was pretty good, and I would prefer them to be out.”

(Int 20; Child: boy, low MVPA, low SED; Parent: female, high MVPA, low SED; IMD score: 44.5)

“When we grew up there was no technology, we had a better quality of life then.”

(Int 11; Child: boy, low MVPA, high SED; Parent: female, low MVPA, low SED; IMD score: 11.9)

“I don’t know how much of it is people romantically thinking about the past, when you used to go out on your chopper for the day and cycle around the city centre and think how wonderful it was, obviously it’s a bit of shame they can’t do that anymore.”

(Int 49; Child: girl, high MVPA, high SED; Parent: male, low MVPA, high SED; IMD score: 8.1)

Parents who perceived their childhood to have been better were more deprived than the overall group, and were representative of the wider group for parent activity, while their children’s activity tended to be classified as ‘high MVPA, high SED’.

## 4. Discussion

This is the first study to explore the views of contemporary UK parents of primary school children in regards to their children’s experiences of physical activity and screen viewing and how they felt this compared to their recall of their own childhood experiences. Compared to the relative freedom they remember enjoying as children, parents commented on how they restrict their children’s independent mobility and outdoor play due to concerns about safety. Previous studies have found that parent’s perceived neighbourhood safety is positively associated with children’s physical activity behaviour [70], and that children whose parents constrict their physical activity due to concerns about neighbourhood safety are less active [35,71]. In the present study, the normalisation of fear was especially prevalent among mothers, although one father did say that by allowing your child to play outside unsupervised you would be ‘considered a bad parent’. This ‘culture of fear’ [27,43,44] is influencing how parents allow their child to interact with their local environment, when in reality these fears of danger outweigh the realities of neighbourhood safety [36,38], with the media blamed for amplifying such fears [72]. Parents discussed how, when they were young, physical activity was just something they did for fun, reminiscing about being sent out to play with other neighbourhood children until it was dark outside. In comparison, parents described their children’s physical activity as being very structured and adult-supervised. They also commented on how children today are much more aware of the value placed on physical activity, in regards to the associated health benefits. The majority of parents perceived their own childhood as better, with several feeling sad that their children will not have the same experiences. Interestingly, parents who reported recreating their own parents’ approach to managing physical activity and screen viewing tended to be more deprived than parents who were actively taking a different approach to managing these behaviours. It was clear that some parents felt it was outside of their control to recreate a similar childhood for their children, in regard to their physical activity. However, a few parents did report setting similar limits for child screen viewing time in relation to the amount of screen viewing they engaged in as children, because they ‘turned out okay’. It is possible that parents feel they can influence discrete behaviours within the home, for example setting screen viewing limits, but outside influences and social norms play a greater role in how they allow their child to be active outside of the home.

Parents commented on the different contexts in which they and their children spent their childhoods (e.g., rural versus urban). A few parents highlighted the environment their child is growing up in as different from their own childhood, and how they believe this may affect their child’s physical activity behaviour. Interestingly, there was little consistency in the type of physical environment with which this observation was noted, with some parents believing that they had more freedom when they were children because they lived in the countryside where there was more space to play. In contrast, other parents believed they did more walking as a child because they lived in the city where they were free to roam, compared to their child who is growing up in the countryside where there is a scarcity of pavements. Therefore, perceptions of the physical environment may be less to do with the specific contexts and more reflective of parents’ safety concerns about the amount of traffic causing both environments to appear more dangerous compared to a generation ago [36,38]. Data from the US National Survey of Children’s Health suggest that families from deprived neighbourhoods are more likely to describe their local area as unsafe [73]. Therefore, it was interesting that, in the present study, the parents who talked about safety concerns, the difference in the built environment, and how this affects their child’s physical activity were representative of the overall sample in terms of deprivation. Therefore, while neighbourhood safety may be of greatest concern for families below the poverty line, it is clearly an issue that affects families across the whole deprivation spectrum. Karsten [46] compared interview data from children from three neighbourhoods in Amsterdam with memories of unrelated people who grew up in these neighbourhoods 50 years previously, to explore perceptions of how these neighbourhoods have changed across generations in relation to children’s outdoor play and independent mobility. The study found that children’s geographies have become more diverse. In addition to the traditional childhood of outdoor children where public street spaces were a child domain, indoor children and children of the ‘backseat generation’ have emerged, characterised by a decrease in playing outdoors and an increase in adult supervision [46]. These results suggest that changes in children’s outdoor play and independent mobility have occurred within neighbourhoods, as well as within families across generations as perceived by parents in the present study.

There is an overall tendency in society to assume things were better in the past [74], with one parent in this study even suggesting that they may be romanticising their childhood (Interview 49). Detailed interview guides with prompts were used to help interviewees to recall all the different aspects of their childhood; however, there is an abundance of research evidencing that people remember the big events and positive experiences (‘go out on your chopper for the day’) whilst neglecting to remember the everyday and less appealing events [46,75]. With this in mind, it needs to be acknowledged that it is unlikely that the generation of children that our study reflects had the freedoms that some of them suggest. Social norms existed in the 1970s, 80s, and early 90s when these parents were children that are likely to have exerted controls on how they spent time outside, both for their pleasure and out of necessity [46].

Screen viewing behaviours are becoming increasingly important for children and adults alike due to the ubiquity of technology in modern society [76]. Parents in the current study, especially those from more deprived neighbourhoods, commented that they felt children have a greater number of distractions now because of the constant availability of children’s television programming, and that advances in technology have led to children becoming ‘lazier’. While parents may experience feelings of internal conflict regarding their child’s screen viewing behaviour [76], children are spending more ‘play’ time indoors engaging in screen viewing [48], partly due to children’s preference for such activities, but also due to the perceived safety of such activities, whereby screen viewing acts as an ‘electronic babysitter’ [77,78]. Therefore, it would be useful for researchers to work with parents and children to develop strategies to support children to reconnect with their neighbourhood environment, engage in unstructured activities close to home, and receive education on how to navigate their home range safely. Pokémon Go is an example of a mobile application that incorporates screen viewing technology, gamification, and outdoor physical activity, and has been associated with short-term (i.e., 30 days) physical activity increases among users [79]. However, more research is needed to translate this into long-term behaviour change. Another example is the ‘Playing Out’ initiative, whereby streets are closed to through traffic for a couple of hours, creating a safe place for children to play. A report by Play England [80] found that children were outdoors for over 70% of the time the streets were closed and spent an average of 16 min per hour in moderate-to-vigorous-intensity physical activity during street closures, but the initiative is limited to individual neighbourhoods and specific time periods each month.

The results from the current study outline key challenges faced by parents when promoting a healthy balance of physical activity and screen viewing for their children, and to what extent these challenges are the same or different from those experienced during their own childhood. Table 2 summarises the key findings and recommendations for how parents can be supported to help their children be physically active and manage screen time. Due to the complexity of the problem (e.g., road safety, urban design, work patterns, child care, economy, media, societal norms), interventions need to be implemented from policy to individual. Therefore, it is important that policy-makers consider these issues when developing policies that may not seem related to childhood independence or parenting practices.

### Strengths and Limitations

The recruitment of a relatively large sample of parents with a good level of variation in socio-economic position is one of the key strengths of the study. The sample includes 20 fathers, a group that are known to be difficult to engage in research [81]. As this qualitative research was embedded within a large cohort study, the sampling process enabled us to explore the experiences of a variety of families with parents and children expressing a range of childhoods. The result is a very rich and unique dataset that has provided novel insights into how childhoods have changed across the last generation, with respect to physical activity and screen viewing. Moreover, the robustness of the data collection and analysis process has provided a rigorous evaluation of the area, and there was a clear saturation of information in the analyses. In addition, the study design allowed for the analysis of the data in the context of parent and child objectively assessed physical activity and sedentary time and a measure of socio-economic status (IMD). The study is, however, limited, as it relied on parents accurately recalling experiences from their own childhood, due to the lack of availability of historical data. Parents were also largely White British (94%) and therefore we have not captured the views of culturally diverse childhoods. Additionally, the study was only conducted in one area in the Southwest of England, and as such the ability to extrapolate to other settings and countries is limited.

## 5. Conclusions

Children’s physical activity and screen viewing behaviours have changed across the last generation. Due to concerns about safety, parents do not allow their children to play outside independently like they used to when they were young. Despite children currently having greater access to structured after-school activities, parents feel that children are missing out, perceiving their own childhood to be better, in part due to the lesser emphasis placed on screen viewing when parents were young. Innovative ways are needed to change the social norms around children’s independent mobility and freedom to play outside.

## Figures and Tables

**Table 1:** Demographic characteristics of the interview sample of parents (*n* = 51) and their children.

	Parents		Children	
	Mean (SD)	%	Mean (SD)	%
Gender (% female)		60.8		51.0
Age (years)	41.2 (4.5)	---	9.0 (0.4)	---
Body mass index (kg/m^2^) *	25.8 (6.1)	---	0.01 (0.95)	---
Index of multiple deprivation	11.5 (9.7)	---	---	---
Moderate-to-vigorous physical activity (mins/day)	48.1 (21.5)	---	58.3 (17.4)	---
Sedentary time (mins/day)	568.3 (149.3)	---	451.9 (103.6)	---
Ethnicity				
White British	---	94.1	---	---
Other	---	5.9	---	---
Employment				
Full-time	---	45.1	---	---
Part-time	---	39.2	---	---
Unemployed/full-time parent	---	15.7	---	---

* Body mass index value for children is the BMI z-score based on the British 1990 Growth Reference [69].

**Table 2:** Key findings and recommendations for behaviour change programmes.

Finding	Recommendation
It is the ‘norm’ for parents, especially mothers, to be fearful about allowing their child to play outside	Improve the way child safety is presented in the media. Encourage families to participate in play street initiatives and share supervision responsibilities
Both rural and urban built environments are not perceived to be as safe as they were a generation ago	Develop policies (e.g., road safety, urban design, education) to improve safety in residential areas
Indoor play (e.g., screen viewing) is seen as a ‘safe’ option	Develop strategies to support children to reconnect with their neighbourhood environment and safely navigate their home range
Parents and children are more aware of the value of physical activity nowadays	Provide parents with ideas and suggestions for how they can be active with their children
Parents who expressed that there is greater provision for children’s activities nowadays tended to be less deprived	Ensure equity in provision of children’s activities across deprivation groups
Parents perceived their childhood to have been better, but feel it is outside of their control to recreate a similar childhood for their children in regard to physical activity	Encourage parents to find small ways give their children more freedom in regard to outdoor play and independent mobility
